# Evaluation of the inner wellbeing model in Zambia

**DOI:** 10.1186/s13612-014-0016-5

**Published:** 2014-08-19

**Authors:** Stanley O Gaines

**Affiliations:** Department of Psychology, Brunel University, Kingston Lane, Uxbridge, UB8 3PH UK

**Keywords:** Confirmatory factor analysis, Inner wellbeing, Zambia

## Abstract

**Background:**

Results of a recently published paper from members of the Wellbeing and Poverty Pathways Project team indicated that a seven-domain, intercorrelated-factor model of economic confidence, agency/participation, social connections, close relationships, physical/mental health, competence/self-worth, and values/meaning as dimensions of inner wellbeing yielded optimal goodness-of-fit (compared to a single-factor model) among a large sample of individuals in rural India. The goal of the present study was to determine whether this model also provided optimal goodness-of-fit among a similarly large sample of individuals in rural Zambia.

**Findings:**

A 35-item survey measuring each of the seven domains of inner wellbeing (5 items per domain) was administered to 344 individuals (140 men, 204 women). Results of confirmatory factor analyses indicated that the seven-factor intercorrelated model not only was acceptable in itself but also yielded significantly better goodness-of-fit than did a one-factor model. Furthermore, 34 of the 35 items loaded significantly and positively on their hypothesised factors.

**Conclusions:**

Overall, results from the present paper – combined with results from the recently published paper by members of the Wellbeing and Poverty Pathways Project team – indicate that the seven-factor intercorrelated model can be generalised across India and Zambia. Implications for studies of wellbeing within (as well as outside) developing nations are discussed.

## Statement of research hypotheses

The goal of the present study was to determine whether results of a recent paper published in *Social Indicators Research* by members of the Wellbeing and Poverty Pathways Project team (White et al. 2012) concerning the goodness-of-fit of a seven-factor, intercorrelated model identifying economic confidence, agency/participation, social connections, close relationships, physical/mental health, competence/self-worth, and values/meaning as domains of *inner wellbeing* (i.e., individuals’ feelings and thoughts about what they can do or be) among a large sample of individuals in rural India can be generalised to a similarly large sample of individuals in rural Zambia. In this brief report, the following hypotheses were tested: (1) A seven-factor, intercorrelated model regarding domains of wellbeing will yield acceptable goodness-of-fit in itself. (2) Furthermore, a seven-factor, intercorrelated model will yield significantly better goodness-of-fit than will a single-factor model.

## Method

### Participants

A total of 370 participants (152 men, 218 women) participated in the present study. The average age of participants was 39.29 years (*SD* = 11.46 years). Data concerning one or more inner wellbeing items were missing for 26 individuals, leaving a final sample of 344 individuals.

## Materials

Individuals completed a 35-item inventory, designed to measure each of the seven domains of wellbeing (5 items per domain) identified by White et al. (2013). Each item was scored according to a 5-point, Likert-type format, with the content of the scale points varying from item to item. Sample items include the following: “How well would you say you are managing economically at present”, measuring economic confidence (1 = very badly, 5 = very well); “If there is a village meeting do you have an opportunity to voice your opinion”, measuring agency/participation (1 = I never get the opportunity to speak, 5 = I always get the opportunity to speak); “Do you know the kind of people who can help you get things done”, measuring social connections (1 = I don’t know anybody at all, 5 = I know people who can get help with whatever I need); “When your mind/heart is troubled/heavy, do you feel there is someone you can go to”, measuring close relationships (1 = never, 5 = always); “Do you ever have trouble sleeping”, measuring physical/mental health (1 = always, 5 = never); “How well have you been able to face life’s difficulties”, measuring competence/self-worth (1 = very badly, 5 = very well); and “How far would you say you feel peace in your heart at the end of the day”, measuring values/meaning (1 = utterly unfair, 5 = totally fair). For each of the items, higher scores reflected higher levels of inner wellbeing within a particular domain.

### Procedure

Prior to conducting the present study, the author obtained institutional ethics approval from The Ethics Review Committee, Department of Psychology, Brunel University. Participation was voluntary; no financial incentives were offered. Participants were recruited via snowball sampling, in which two or more members of the author’s research team (at least one of whom usually was a local resident and was fluent in the local language) approached households in a rural area within Chiawa, Zambia. Each participant provided informed consent orally. In turn, each participant gave oral responses to a survey that contained the aforementioned inner wellbeing items and several additional questionnaires that will not be discussed further. Finally, each participant received oral debriefing from two or more members of the research team (at least one of whom usually was a local resident and was fluent in the local language).

## Results

Following the calculation of an item correlation matrix (available from the author upon request and generated via PRELIS 9.1; Joreskog & Sorbom 2012b), results of confirmatory factor analyses with maximum likelihood solutions, ridge options, and ridge constants (conducted via LISREL 9.1; Joreskog & Sorbom 2012a), indicated that (consistent with hypotheses), a seven-factor, correlated model provided adequate fit to the correlational data (chi-square = 231.06, degrees of freedom = 567, *NS*; chi-square/degrees-of-freedom ratio = .41; standardised root mean square residual = .03; adjusted goodness-of-fit index = .96). Moreover, all loadings were positive; and significant loadings emerged for 34 of the 35 items on their respective factors (i.e., with the exception of the values/meaning item “To what extent have you been able to practice your religion in the way you would like”, *p*’s for factor loadings < .05 or lower; details regarding items and factor loadings within each of the domains of inner wellbeing are available from the author upon request). Finally (and consistent with hypotheses), the seven-factor intercorrelated model provided a significantly better fit to the correlational data than did the one-factor model (reduction in chi-square from one-factor model to seven-factor intercorrelated model = 58.90; reduction in degrees of freedom from one-factor model to seven-factor intercorrelated model = 27, *p* < .01). One caveat concerning internal consistency: Even taking into account the relatively small number of items per scale (see Nunnally & Bernstein 1994), reliabilities were somewhat low across the scales (average Cronbach’s alpha = .55; compare to the average Cronbach’s alpha of .61 that White et al. 2013, reported for their Time 2 sample in India). Nevertheless, results of the present study indicate that the seven-factor intercorrelated model that White et al. (2013) reported as yielding optimal fit for data among participants in rural India also yielded optimal fit for data among participants in rural Zambia. Although the seven-factor intercorrelated model should also fit data for individuals in developed nations (at least in principle), further work must be done concerning the generalisability of the model across individuals in developing and developed nations alike (see also White et al. 2012). Nevertheless, results of White et al. (2013) and of the present study suggest that, at least among individuals in two developing nations, the seven-factor intercorrelated model of domains of inner wellbeing is valid.
